# Preclinical validation of the small molecule drug quininib as a novel therapeutic for colorectal cancer

**DOI:** 10.1038/srep34523

**Published:** 2016-10-14

**Authors:** Adrian G. Murphy, Rory Casey, Aoife Maguire, Miriam Tosetto, Clare T. Butler, Emer Conroy, Alison L. Reynolds, Kieran Sheahan, Diarmuid O’Donoghue, William M. Gallagher, David Fennelly, Breandán N. Kennedy, Jacintha O’Sullivan

**Affiliations:** 1 Centre for Colorectal Disease, St Vincent’s University Hospital, Elm Park, Dublin 4, Ireland; 2 UCD School of Biomolecular and Biomedical Science Conway Institute, University College Dublin, Dublin 4, Ireland; 3 Trinity Translational Medicine Institute, Department of Surgery, Trinity College Dublin, St James’s Hospital, Dublin 8, Ireland; 4 Department of Histopathology, St. James’s Hospital, Dublin 8, Ireland

## Abstract

Colorectal cancer (CRC) is a leading cause of cancer deaths. Molecularly targeted therapies (*e.g*. bevacizumab) have improved survival rates but drug resistance ultimately develops and newer therapies are required. We identified quininib as a small molecule drug with anti-angiogenic activity using *in vitro*, *ex vivo* and *in vivo* screening models. Quininib (2-[(*E*)-2-(Quinolin-2-yl) vinyl] phenol), is a small molecule drug (molecular weight 283.75 g/mol), which significantly inhibited blood vessel development in zebrafish embryos (p < 0.001). *In vitro*, quininib reduced endothelial tubule formation (p < 0.001), cell migration was unaffected by quininib and cell survival was reduced by quininib (p < 0.001). Using *ex vivo* human CRC explants, quininib significantly reduced the secretions of IL-6, IL-8, VEGF, ENA-78, GRO-α, TNF, IL-1β and MCP-1 *ex vivo* (all values p < 0.01). Quininib is well tolerated in mice when administered at 50 mg/kg intraperitoneally every 3 days and significantly reduced tumour growth of HT-29-luc2 CRC tumour xenografts compared to vehicle control. In addition, quininib reduced the signal from a α_v_β_3_ integrin fluorescence probe in tumours 10 days after treatment initiation, indicative of angiogenic inhibition. Furthermore, quininib reduced the expression of angiogenic genes in xenografted tumours. Collectively, these findings support further development of quininib as a novel therapeutic agent for CRC.

Colorectal cancer (CRC) is the leading cause of cancer deaths in the Western world with over 300,000 new cases of the disease diagnosed per year and an annual mortality of approximately 130,000^1^. The development of molecularly targeted agents, specifically anti-angiogenic drugs, has improved survival in CRC^2^,^3^. Bevacizumab (Avastin) improves overall survival in CRC by 23% when combined with cytotoxic chemotherapy although ~55% of patients do not respond to bevacizumab^4^. In addition, regorafenib was the first small molecule anti-angiogenic drug to be licensed for use in CRC^5^. Despite these advances, tumours ultimately become resistant to these agents therefore the development of novel anti-angiogenic agents is required.

*In vitro* drug discovery screening models lack any resemblance of the tumour microenvironment which is important for modelling tumour behaviour. In contrast, *in vivo* models retain the physiological complexity between a tumour and the whole-organism system. Zebrafish (*Danio rerio*) are increasingly being used as a vertebrate model organism for cancer research^6^. Advantages include their small size, low costs of maintenance, high rates of fecundity and the availability of transgenic strains. Tg[*fli1*:EGFP] zebrafish drive EGFP (enhanced green fluorescent protein) expression using the *fli1* promoter and have been used extensively in anti-angiogenic drug discovery^7^,^8^. The *fli1* promoter is the earliest endothelial cell marker in zebrafish which persists in blood vessels until 2 days post fertilisation (dpf) and EGFP-tagged blood vessels are easily visualised using fluorescence microscopy^9^. Chemical screens in zebrafish larvae have previously detected promising novel compounds with anti-cancer activity^10^,^11^,^12^. White *et al*. identified leflonamide, a dihydrorotate dehydrogenase inhibitor which affected cell cycle activity in zebrafish and showed that it reduced melanoma development when combined with BRAF inhibition in a preclinical murine model^13^. Unbiased zebrafish drug screens allow exploration of the “chemical space” to detect compounds with previously unknown anti-angiogenic activity^14^.

In this study, we demonstrate that a small molecule inhibitor quininib has robust anti-angiogenic and anti-tumour efficacy using *in vitro*, *ex vivo* and *in vivo* models of colorectal cancer.

## Results

### Unbiased chemical screening with zebrafish larvae detects novel anti-angiogenic agent, quininib

**Figure 1:**
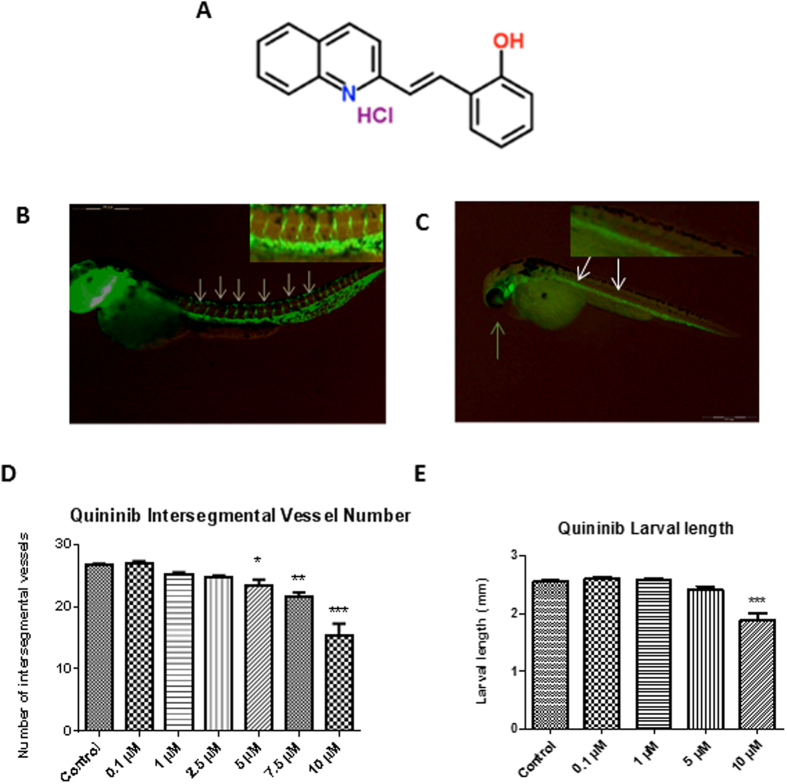
Quininib reduces intersegmental blood vessel formation in zebrafish larvae. (**A**) Chemical structure of 2-[(*E*)-2-(Quinolin-2-yl) vinyl] phenol (quininib). (**B,C**) Representative images of larvae treated with vehicle control (**B**) and 10 μM quininib (**C**). The white arrows in (**C**) demonstrate the absence of ISV and the green arrow shows the presence of pericardial oedema and magnified views of ISVs are inset in the right upper corner of each panel. (**D**) Quininib reduced ISV number at 5 μM (12%), 7.5 μM (18.8%) and 10 μM concentration (33%). (**E**) Quininib reduced larval length at 10 μM. Error bars are mean + SEM. Statistical analysis performed by ANOVA and Dunnett’s multiple comparison test. N = 3 experiments with 10 embryos per concentration *p < 0.05, **p < 0.01, ***p < 0.001.

An unbiased phenotype-based screen of 1000 chemicals from a subset of the Chembridge DIVERSet library revealed quininib as the lead hit in intersegmental developmental angiogenesis assays (Fig. 1A). Quininib (or 2-[(*E*)-2-(Quinolin-2-yl)vinyl]phenol ) is a small molecule compound which contains a benzene ring fused to a quinoline ring^12^.

In our studies, quininib was shown to elicit the greatest effect at 10 μM, reducing intersegmental vessel (ISV) development by 67% (p < 0.001, Fig. 1D). Larvae treated with 10 μM quininib were noted to have pericardial oedema (defined as excess fluid in the region of the developing heart) and found to be 23% shorter at 48 hpf (Fig. 1E) whereas no larvae treated with vehicle control displayed these phenotypes. Larvae treated with quininib did not exhibit abnormal locomotor behaviours and no additional morphological abnormalities were observed.

### Quininib reduces endothelial tubule formation and cancer cell survival

**Figure 2:**
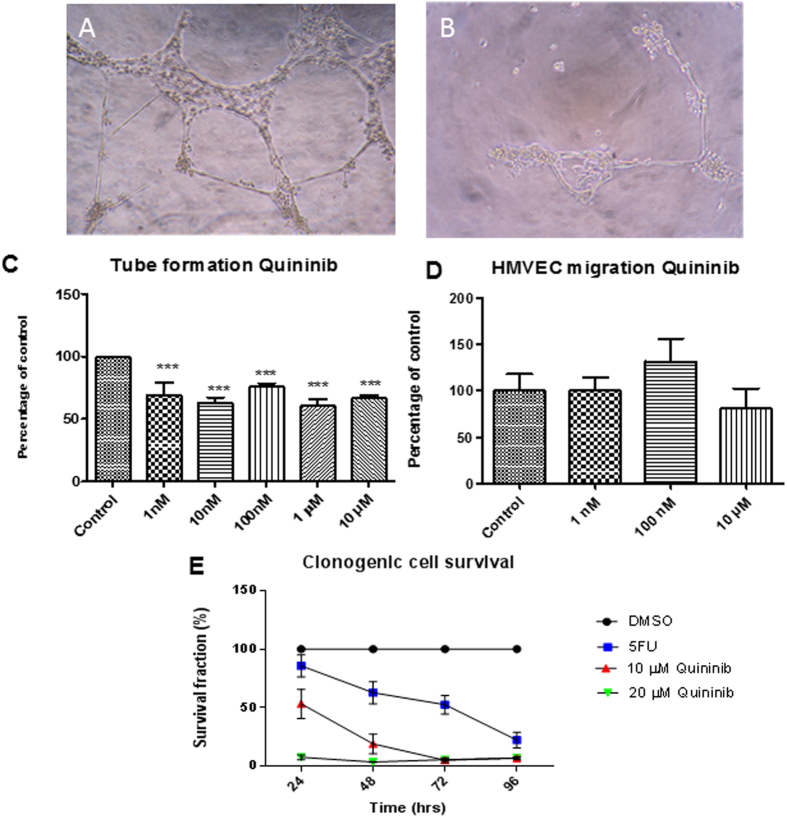
Quininib reduces endothelial tubule formation and cancer cell proliferation. (**A**) Representative image showing HMVEC-D tubule formation when treated with vehicle control. (**B**) Representative image showing reduced HMVEC-D tubule formation when treated with 10 μM quininib. (**C**) Quantification of tubule formation reduction is detected between 1 nM–10 μM quininib. (**D**) Quantification of HMVEC-D migration shows quininib does not alter migration (1 nm–10 μM). (**E**) Clonogenic assay shows reduced cell survival with 10 μM and 20 μM quininib (p < 0.0001). Error bars are mean ± SEM. Statistical analysis performed by ANOVA and Dunnett’s multiple comparison test. N = 3 experiments. ***p < 0.001.

In dermal-derived human microvascular endothelial cells HMVEC-D, concentrations of 1 nM to 10 μM quininib reduced tubule formation, (p < 0.001) (Fig. 2C). The migration rate of HMVEC-Ds was not affected by 10 μM quininib (Fig. 2D). Using clonogenic assays, 10 μM and 20 μM quininib significantly reduced cell survival (p < 0.0001) compared to vehicle control (Fig. 2E).

### Quininib reduces VEGF, IL-6 and IL-8 secretion in a human CRC explant model

**Figure 3:**
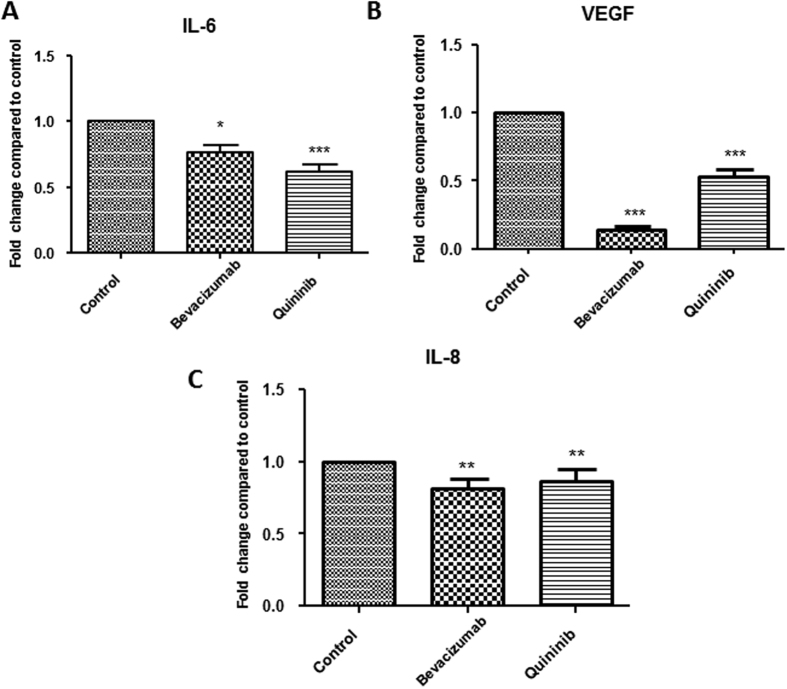
Quininib reduces the secretion of angiogenic growth factors and inflammatory cytokines in an explant model of colorectal cancer. Colorectal cancer *ex vivo* human explants were treated with either 10 μg/ml bevacizumab or 10 μM quininib for 72 hours. The levels of IL-8, VEGF and IL-6 were measured by ELISA and normalised to explant protein content. Levels of IL-8 (**A**), VEGF (**B**) and IL-6 (**C**) were significantly reduced following both bevacizumab and quininib treatments. Error bars are mean + SEM. Statistical analysis performed by Wilcoxon signed rank test. *p < 0.05, **p < 0.01, ***p < 0.001.

We measured the effects of quininib on the protein secretions of angiogenic growth factors and inflammatory cytokines in human *ex vivo* colorectal cancer explants from 40 patients (10 per stage) with CRC. Treatment with 10 μM quininib resulted in significant reduction in secretions of important angiogenic mediators IL-6 (37.8%), VEGF (47.3%) and IL-8 (13.2%), (Fig. 3A–C respectively). Treatment with 10 μg/ml bevacizumab also resulted in the reduced secretion of IL-8, IL-6 and VEGF. The secretion of other mediators including ENA-78, GROα, MCP-1, TNF and IL-1β were also significantly reduced following quininib treatment (all p < 0.01, Supplementary Fig. S1). Quininib elicited similar effects in decreasing the above analytes across all stages of CRC (stages I–IV).

### Quininib reduces tumour growth and bioluminescence in a colorectal cancer mouse model

**Figure 4:**
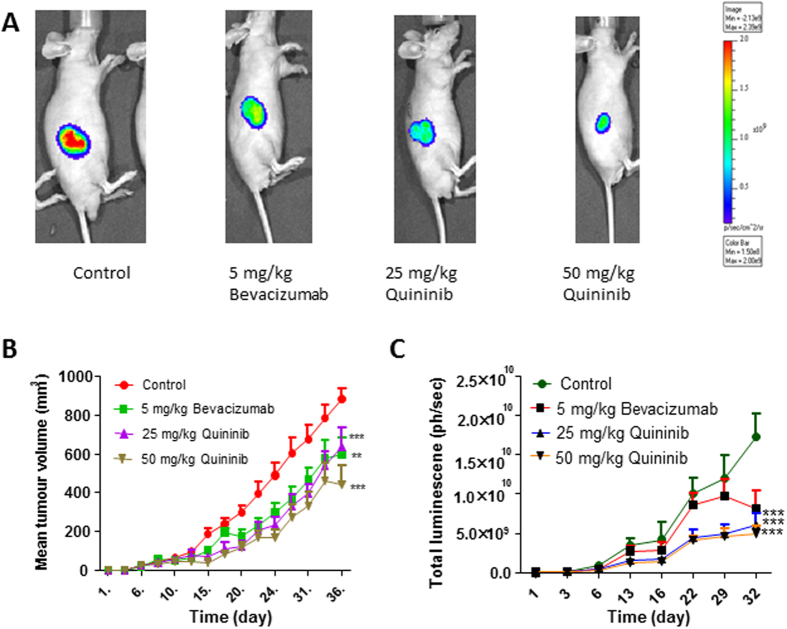
Quininib reduces tumour growth in a xenograft model of colorectal cancer. (**A**) Representative images of bioluminescent images taken from animals treated with vehicle control, 5 mg/kg bevacizumab, 25 mg/kg quininib and 50 mg/kg quininib. (**B**) Graph of tumour volume (mm^3^) shows reduced tumour development in 5 mg/kg bevacizumab, 25 mg/kg quininib and 50 mg/kg quininib compared to control. (**C**) Graph showing reduced bioluminescent activity in 5 mg/kg bevacizumab, 25 mg/kg quininib and 50 mg/kg quininib compared to control. Error bars are mean + SEM. Statistical analysis performed by ANOVA and Dunnett’s multiple comparison test. **p < 0.01, ***p < 0.001.

We created a xenograft model of colorectal cancer by injecting 2.5 × 10^6^ HT-29-luc2 cells into Balb/C nu/nu mice. When tumours reached 100 mm^3^, we randomised the animals into treatment groups comprising 5 mg/kg bevacizumab, 25 mg/kg quininib or 50 mg/kg quininib. We measured tumour growth using calliper measurements and optical imaging (bioluminescence, BLI). Treatment with 50 mg/kg quininib resulted in significant decrease in tumour volume, as measured using callipers, compared to control (average final measurements: 442.1 mm^3^ vs. 881.6 mm^3^, p < 0.001, Fig. 4B). Treatment with 5 mg/kg bevacizumab and 25 mg/kg quininib also significantly reduced tumour volume compared to control (average final measurements: 559.3 mm^3^ and 639.8 mm^3^ respectively, p < 0.001). Treatment with 50 mg/kg quininib did not significantly reduce tumour volume compared to 5 mg/kg bevacizumab or 25 mg/kg quininib. Similarly, BLI signals were lowest in the 50 mg/kg quininib group compared to control (p < 0.001, Fig. 4C). BLI was also lower in the 25 mg/kg quininib and 5 mg/kg bevacizumab groups (all p < 0.001, Fig. 4B) and was not significantly different from 50 mg/kg quininib.

### Quininib reduces angiogenic signalling in a xenograft model

**Figure 5:**
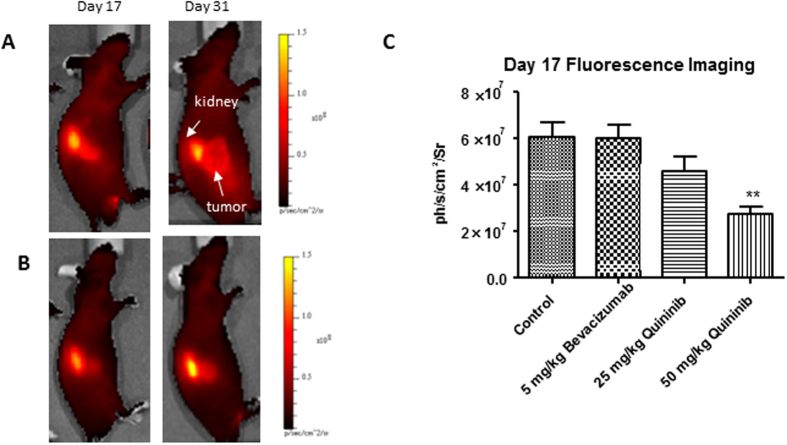
Quininib reduces angiogenesis in a xenograft model of colorectal cancer. (**A**) Representative images of fluorescence of α_v_β_3_ integrin-bound probe in animals treated with vehicle control at days 17 and 31. White arrows highlight kidney and tumour (**B**) Representative images of animals treated with 50 mg/kg quininib at days 17 and 31. (**C**) Reduced fluorescence at day 17 in the 50 mg/kg quininib group. Graphed is mean + SEM. Statistical analysis performed by ANOVA and Dunnett’s multiple comparison test **p < 0.01.

We used a fluorescent probe which binds to α_v_β_3_ integrin *in vivo* and acts as a surrogate of tumour-associated angiogenesis. This probe was injected on days 17 and 31 of the study (10 and 24 days after initiation of treatments) and fluorescence was measured 4 hours after injection. We found that fluorescence was significantly reduced in the 50 mg/kg quininib (p < 0.01) compared to control on day 17 (Fig. 5A–C). Fluorescence was not significantly reduced in the 5 mg/kg bevacizumab or 25 mg/kg quininib groups on day 17 (Fig. 5C). When we repeated fluorescent imaging on day 31 (24 days after the initiation of treatment), there were no significant differences in any of the groups compared to control (Supplementary Fig. S2).

### Quininib reduces angiogenic gene expression in xenografts

Gene expression changes in HT-29-luc2 cells treated with bevacizumab or quininib were screened using the RT^2^ profiler human angiogenesis array. Of the 84 genes analysed, quininib altered the expression of 31 genes (37%) and bevacizumab altered 17 genes (20%) (Supplementary Fig. S3).

**Figure 6:**
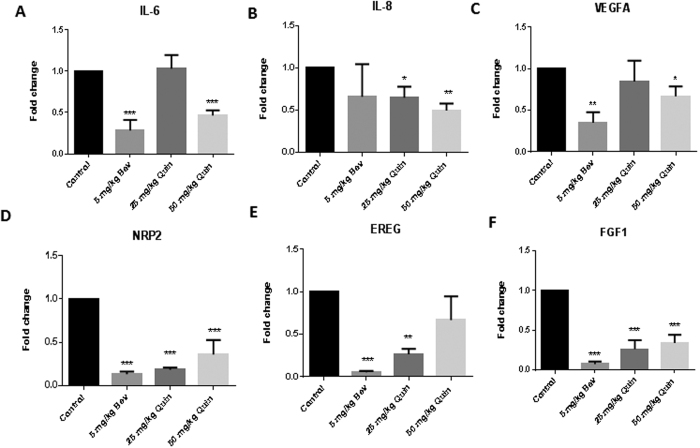
Quininib reduces angiogenic gene expression in HT-29luc2 xenografts. Changes in gene expression of *IL-6* (**A**), IL-8 (**B**), *VEGFA* (**C**), *NRP2* (**D**), *Epiregulin* (**E**) and *FGF1* (**F**) were measured by RT-PCR. Error bars graphed are mean + SEM. Statistical analysis was performed by ANOVA and Dunnett’s multiple comparison test. *p < 0.05, **p < 0.01, ***p < 0.001. (Bev: Bevacizumab, Quin: Quininib).

We examined the expression of genes closely associated with angiogenesis in CRC xenografts by quantitative PCR. Quininib treatment (50 mg/kg) resulted in the reduced expression of interleukin-6 (*IL6*), interleukin-8 (*IL8*), vascular endothelial growth factor A (*VEGFA*), neuropilin-2 (*NRP2*) and fibroblast growth factor 1(*FGF1*) (Fig. 6A–F). Bevacizumab treatment (5 mg/kg) resulted in the reduced expression of *IL6*, *IL8*, *VEGFA*, *NRP2*, *EREG* (epiregulin) and *FGF1*.

## Discussion

We conducted a chemical screen which detected quininib, a compound with anti-angiogenic activity in zebrafish embryos. *In vitro*, quininib reduced both survival of HT-29-luc2 CRC cells and endothelial cell tubule formation but did not reduce HMVEC-D migration rate. We cultured human colorectal tumours *ex vivo* and found that the secretion of different angiogenic and inflammatory mediators were reduced by quininib. Quininib reduced tumour growth and bioluminescent activity compared to vehicle control in a CRC xenograft model. In addition, α_v_β_3_ integrin fluorescent probe signal was reduced by quininib ten days after initiation of treatment. We further demonstrated the anti-angiogenic capacity of quininib by examining gene expression of angiogenic factors in xenograft tumours and found that the expression of *IL6*, *IL8*, *NRP2*, *VEGFA* and *FGF1* was reduced by quininib treatment.

Quininib, a quinoline, detected in an unbiased small molecule chemical screen, reduced intersegmental vessel formation in Tg [*fli1*: EGFP] zebrafish embryos, a transgenic model allowing easy visualization of developing vasculature^7^,^15^,^16^,^17^. We observed reduced larval lengths when embryos were treated with 10 μM quininib at 48 hpf. This effect has been observed in other reported screens of anti-angiogenic agents in zebrafish where compounds were added between 6–10 hpf, and is regarded as an expected effect of inhibiting angiogenesis, rather than a toxic effect^18^,^19^. Zebrafish embryos undergo considerably rapid morphogenetic processes up to 48 hpf and small molecule screens have to capacity to detect system-specific toxicities as early as 24 hpf^8^,^20^. Aside from pericardial edema, another recognised anti-angiogenic effect, larvae treated with quininib did not demonstrate any additional behavioural or morphological deficits, indicating that quininib is non-toxic. Previously, using a zebrafish screening approach, Ewan *et al*. identified rosuvastatin, a small molecule inhibitor of Wnt signalling exhibiting anti-angiogenic and anti-tumour properties in prostate cancer^21^,^22^. This study validates the use of zebrafish in screening anti-angiogenic compounds due to the high functional conservation between zebrafish and human vascular development^23^,^24^.

Quininib did not affect endothelial cell migration rate but did reduce tubule formation in human endothelial cells although it possible that higher concentrations may exert a dose dependent effect. However, 10 μM quininib was sufficient to produce anti-angiogenic effects in multiple models including zebrafish and human *ex vivo* explants. In preclinical studies, bevacizumab reduces human endothelial cell VEGF_165_ – induced growth and migration when stimulated with the specific ligand of this monoclonal antibody, and treatment with bevacizumab in the absence of VEGF_165_ –stimulation does not reduce endothelial cell growth or migration^25^,^26^. The tubule formation assay, as a complex multi-step process, represents the reorganization stage of physiological angiogenesis^27^,^28^. It is, therefore, a superior *in vitro* functional assay to test novel anti-angiogenic compounds.

Regorafenib, a multi-tyrosine kinase inhibitor known to prolong survival in metastatic colorectal cancer, reduces the cell proliferation rate of a variety of cancer cell lines *in vitro* including a potent effect on a GIST (gastrointestinal stromal tumour) cell line with only a modest effect on a CRC cell line^5^,^29^. This may be explained by the relative sensitivities of selected cell lines to certain therapeutic agents or it may highlight the discrepancies that exist between *in vitro* assays and clinical findings.

We used human CRC explants to assess quininib’s effect on the secretion of angiogenic and inflammatory factors as this model has previously detected abundant secretion of these factors in the colorectal tumour microenvironment^29^,^30^,^31^,^32^. In this study, the secretion of important angiogenic and inflammatory mediators were significantly reduced to different levels when *ex vivo* explants were treated with quininib. In addition, all data were normalized to individual explant protein content thereby accounting for any differences in cell content in the explant tissues. Overall, this indicates that these inhibitory effects were not simply due to reduced tissue size or reduced cellular content.

Explants contain the key components of the tumour microenvironment including endothelial and inflammatory cells which engage in complex crosstalk resulting in a balance favouring/opposing angiogenic activity. Bevacizumab affects serum levels of angiogenic growth factors/cytokines and serum levels of IL-6, IL-8, IL-10, epidermal growth factor and macrophage-derived chemokine may predict clinical responses in patients with metastatic colorectal cancer^33^. Given the economic costs of administering anti-angiogenic agents, much interest lies in identifying biomarkers which could detect those who might benefit from anti-angiogenic therapies^34^,^35^.

We used a xenograft CRC model to demonstrate that quininib decreased tumour growth and bioluminescent activity *in vivo*. Preliminary studies had shown that quininib was well tolerated and 50 mg/kg was the maximum tolerated dose (MTD, data not shown). There was no statistical difference between the effects of the MTD and 50% MTD doses on tumour growth and it is possible that lower doses may have a similar effect. We compared the MTD of quininib with the standard dose of 5 mg/kg bevacizumab used in xenograft studies as the MTD of bevacizumab has not been reported^36^,^37^,^38^. The approval of bevacizumab used in combination with cytotoxic chemotherapy in metastatic CRC has led to prolonged overall survival^4^,^39^. It is possible that a further synergistic effect on tumour growth could be found if quininib was combined with a fluoropyrimidine-based chemotherapy regimen.

To date, regorafenib is the only small molecule anti-angiogenic drug licensed for use in treatment-refractory metastatic CRC^5^. However, despite the modest survival advantage of regorafenib compared to placebo, it is not prescribed universally by oncologists due its toxicity profile, therefore there is a role for novel anti-angiogenic agents in this setting^40^.

In order to examine the specific effects of quininib on angiogenesis *in vivo*, we used a fluorescent probe specific for α_v_β_3_ integrin. This integrin is a receptor for extracellular matrix proteins and was the first integrin discovered to regulate angiogenesis^41^. Angiogenic activity was reduced after 10 days of treatment with 50 mg/kg quininib although this anti-angiogenic effect was not seen in those animals treated with bevacizumab. This conflicts with prior data where fluorescence from a probe bound to α_v_β_3_ integrin was shown to be reduced at 7 days with bevacizumab treatment, however, alternative probe/imaging techniques were used^42^. It is possible that quininib and bevacizumab may have different effects on α_v_β_3_ integrin binding or that timing of 7 vs. 10 days is important in this process.

To correlate with our *in vitro* and *ex vivo* data, we examined the effects of quininib on angiogenic gene expression in xenografts. These genes (*IL6*, *IL8*, *NRP2*, *FGF1, VEGFA*) are renowned for their importance in angiogenesis and we found that their expression was reduced by quininib^43^,^44^,^45^,^46^. Using target profiling, quininib has been identified as a cysteinyl leukotriene receptor 1 and receptor 2 antagonist^47^. Although predominantly known for their role in inflammation, cysteinyl leukotrienes have been reported to have pro-angiogenic activities^48^,^49^,^50^.

Tumours ultimately become resistant to anti-angiogenic therapies^51^, therefore, novel anti-angiogenic compounds are needed. In summary, we have identified a novel small molecule anti-angiogenic inhibitor, quininib, which shows activity *in vitro*, *ex vivo* and *in vivo* in CRC models. Future work should focus on identifying additional anti-tumoral effects in combination with chemotherapeutic agents and radiation.

## Methods

### Chemical library

Chemicals for screening were purchased from ChemBridge DIVERSet library (California). All compounds were screened at 10 μM concentration diluted in embryo medium to a final concentration of 0.1% DMSO.

### Zebrafish husbandry

Zebrafish (*Danio rerio*) experiments were performed in accordance with local guidelines (Dept. Health and Children, Ireland). All experimental protocols were approved by the Animal Research Ethics Committee in University College Dublin. Zebrafish were maintained according to standard procedures at 28.5 °C on a 14 hr light/10 hr dark cycle. Embryos were obtained by natural spawning and developmental stages established by time and morphological criteria. Transgenic zebrafish Tg [*fli1*: EGFP] were used for all experiments.

### Intersegmental vessel assay

At 6 hours post fertilisation (hpf) prior to vasculogenesis, 5 embryos (chorionated) were incubated with an individual DIVERSet compound at 10 μM concentration in each well of a 48 well plate with 0.1% DMSO. At 48 hpf, treated larvae were dechorionated, fixed in 4% paraformaldehyde overnight at 4 °C, washed with PBS and assessed for morphological defects including pericardial oedema using brightfield microscopy. Dose response relationship experiments were performed with 0.1, 1, 2.5, 5, 7.5 and 10 μM of relevant compound with at least 30 larvae tested per concentration.

### Imaging

Brightfield and fluorescent images were taken using an Olympus SZX16 stereo zoom microscope with an Olympus DP71 camera. The larval lengths were calculated from brightfield whole larval images using Cell^F software (Olympus) and intersegmental vessels were manually counted at high magnification when Tg [*fli1: EGFP]* larvae were imaged using EGFP excitation/emission filters.

### Cell culture

HMVEC-D (dermal derived human microvascular endothelial cells, authenticated with alpha actin staining by Clonetics, San Diego, USA) were maintained in complete endothelial cell medium (Clonetics^®^ EGM^®^-2-MV Bulletkit^®^) containing 500 ml of Endothelial Cell Basal Medium-2 supplemented with 0.5 ml hEGF, 0.2 ml hydrocortisone, 0.5 ml GA-1000, 25 ml FBS, 0.5 ml VEGF, 2 ml hFGF-B, 0.5 ml R3-IGF-1, 0.5 ml ascorbic acid in a 37 °C humidified atmosphere of 5% CO_2_. HMVEC-D cells were used for experiments between passages 4–8.

### Clonogenic assay

The HT-29-luc2 Bioware^®^ Ultra cell line was purchased from PerkinElmer (UK) and maintained in McCoy’s 5A media with L-glutamine (Fisher) supplemented with 10% fetal calf serum. Cells were washed in DPBS and trypsinised using 2 ml TrypLE Express (1X) (Invitrogen). 1.5 × 10^3^ cells were seeded per well of a 6 well plate and incubated for 24 hours. Cells were then treated with 10 or 20 μM of quininib, 5-fluorouracil (5FU) or 0.1% DMSO vehicle for 24, 48, 72 and 96 hours. The drugs were removed and cells were allowed to grow in fresh media for 10 days in total. Clones were then fixed using 4% paraformaldehyde and stained with 0.5% crystal violet solution (Pro-Lab diagnostics PL.7000) at RT for 2 hours. Clone counting was performed using the ColCount system (Oxford Optronix).

### Tubule formation assay

Matrigel (BD sciences) basement membrane matrix was plated in 96 well plates after thawing on ice and allowed to polymerise at 37 °C in 5% CO_2_ humidified atmosphere for 1 hour. HMVEC-D cells were trypsinised and resuspended in EGM growth medium. Cells were seeded at a density of 5 × 10^5^ cells/cm^2^ overnight and then incubated in the presence of quininib for 30 hours at 37 °C in 5% CO_2_ humidified atmosphere. Endothelial cell tubule formation was assessed using Olympus CKX41 inverted phase contrast microscopy and photographed (ImageJ software). The tubule analysis was determined from 3 sequential fields (20X magnification) with a connecting branch between two discrete endothelial cells defined as one tube.

### Explant processing and culture

Explants were obtained from the Department of Histopathology, St Vincent’s University Hospital, Dublin. All experimental protocols were approved by the St Vincent’s University Hospital, Dublin ethics committee and experimental protocols conducted in accordance with approved guidelines (Irish Council for Bioethics). A piece of colorectal tumour was taken following surgery and stored in a tissue wash buffer which contained PBS supplemented with 4 μg/ml amphotericin B (Invitrogen, Paisley, UK), 100 U/ml penicillin, 100 μg/ml streptomycin and 30 μg/ml gentamicin. Explant tissue pieces from each of the tumours were cut into smaller pieces and placed in pre-warmed RPMI media (supplemented with 20% FCS, 4 μg/ml amphotericin B, 100 U/ml penicillin, 100 μg/ml streptomycin, 30 μg/ml gentamicin) and incubated at 37 °C and 5% CO_2_ for 24 hours. The media was replaced with fresh media containing 0.1% DMSO (control), 100 μg/ml bevacizumab or 10 μM quininib solutions and cultured for 72 hours.

### ELISA (Enzyme Linked ImmunoSorbent Assay)

Tumour conditioning media (TCM) was removed from explant culture and the secretion of cytokines and angiogenic growth factors analyzed by ELISA as per the manufacturers’ instructions. To assess cytokine release, a multiplex kit was used for IL-6, TNF, IL-1β and IL-8 (MSD). Individual ELISA kits were used to assess VEGF, MCP-1, ENA-78 and GROα (R&D Duoset systems). Secretion data for all factors were normalised by explant protein content using the BCA assay (Pierce).

### Xenograft experiments

Animal experiments were performed in accordance with local guidelines (Dept. Health and Children, Ireland). All experimental protocols were approved by the Animal Research Ethics Committee in University College Dublin. All animals were obtained from Harlan, UK aged 4–6 weeks and acclimatised for 7 days prior to experimental use. Animals were monitored and scored daily for physical/behavioural changes during the studies. Animals were fed autoclaved water and irradiated food *ad libitum* and maintained on a 12 hour light/dark cycle. Male athymic mice (Balb/C *nu*/*nu*) were subcutaneously injected into the right flank with 2.5 × 10^6^ cells in PBS/matrigel. Animals were optically imaged 1 hour post injection and twice weekly during the study. Tumours were measured using digital callipers three times weekly and tumour volumes were calculated according to the following formula:


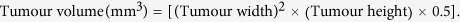


Animals were randomised to receive treatment when tumour volumes reached 100 mm^3^. Animals received vehicle control, 25 mg/kg quininib or 50 mg/kg quininib intraperitoneally every 3 days for a maximum of 30 days. Quininib was dissolved in a 6% ethanol/cremaphor solution (Sigma). Animals received clinical-grade 5 mg/kg bevacizumab twice weekly intraperitoneally for a maximum of 30 days^52^,^53^. Animals were euthanised if tumours became ulcerated or if there was weight loss >20% in one week.

### Optical imaging

Optical imaging was performed using an IVIS spectrum imaging system (PerkinElmer). Animals were anaesthetised by induction with 4–5% isoflurane (Vetflurane, Virbac, UK) and maintained with 1–2% isoflurane during imaging. For bioluminescence studies, luciferin (150 mg/kg, PerkinElmer) was administered intraperitoneally to non-anaesthetised animals 10 minutes prior to imaging. For fluorescence studies, XenoLight RediJect Integrin 750 Probe (2 nmol/100 μL, PerkinElmer) was administered via tail vein injection to anaesthetised animals 10 and 24 days post commencement of treatments. Excitation and emission wavelengths of 745 nm and 820 nm respectively, were used for the acquisition of the *in vivo* fluorescent images. Images and quantitative measurements of bioluminescent and fluorescent signals were obtained and analyzed using Living Image Software v3.2 (Caliper).

### PCR profiler array and Quantitative PCR

RNA was extracted from tumours using TriReagent (Molecular Research Center, Ohio, USA). cDNA was synthesised using reverse transcriptase enzyme and buffer (Bioline, Kilkenny, Ireland) and random primers (Invitrogen. CA, USA). The RT^2^ profiler Human Angiogenesis PCR array (Super Array Bioscience Corp, USA) was used to analyse the expression of 84 genes related to angiogenesis in HT-29-luc2 cells treated with 100 μg/ml bevacizumab or 10 μM quininib. Real time PCR was performed in triplicate samples on ABI Prism 6500 (ABI Biosystems, CA, USA) for 40 cycles and the threshold cycle (Ct) calculated for each sample relative to the 18s ribosomal RNA control expression. Specific primers were used for *VEGFA* (Hs00900055_m1), *EREG* (Hs00914313_m1), *NRP2* (Hs00187290_m1), *FGF1* (Hs00265254_m1), *IL6* (Hs00985639_m1) and *IL8* (Hs01567912_m1) (ABI Biosystems, CA, USA).

### Statistical analysis

Statistical analyses were performed using GraphPad Prism for Windows (version 5.01). Differences between means from multiple groups were assessed by using one way analysis of variance (ANOVA) with the posthoc Dunnett’s multiple comparison test to look for individual differences compared to the control group. For bioluminescence measurements comparing two groups, Mann-Whitney U test was used. Paired data were compared using Wilcoxon signed rank test. All error bars reflect the standard error of the mean. Statistical significance of p < 0.05 is represented as *p < 0.01 as ** and p < 0.001 as ***.

## Additional Information

**How to cite this article**: Murphy, A. G. *et al*. Preclinical validation of the small molecule drug quininib as a novel therapeutic for colorectal cancer. *Sci. Rep.* **6**, 34523; doi: 10.1038/srep34523 (2016).

## Supplementary Material

Supplementary Information
