# Supplementary material for: Preclinical validation of the small molecule drug quininib as a novel therapeutic for colorectal cancer
Source: Sci Rep. 2016 Oct 14;6:34523. doi: 10.1038/srep34523 (PMC5064353; doi:10.1038/srep34523)
Supplement: Supplementary Information [file srep34523-s1.doc]

**Title**: **Preclinical validation of the small molecule drug quininib as a novel therapeutic for colorectal cancer.**

Adrian G Murphy^1, 2^, Rory Casey^3^, Aoife Maguire^4^, Miriam Tosetto^1^, Clare T Butler^2^, Emer Conroy^2^, Alison L Reynolds^2^, Kieran Sheahan^1^, Diarmuid O’Donoghue^1^, William M. Gallagher^2^, David Fennelly^1^, Breandán N Kennedy^2^ & Jacintha O’Sullivan^3*^.

Affiliations:

1. Centre for Colorectal Disease, St Vincent’s University Hospital, Elm Park, Dublin 4, Ireland.

2. UCD School of Biomolecular and Biomedical Research, Conway Institute, University College Dublin, Dublin 4, Ireland.

3. Trinity Translational Medicine Institute, Department of Surgery, Trinity College Dublin, St James’s Hospital, Dublin 8, Ireland.

4. Department of Histopathology, St. James’s Hospital, Dublin 8, Ireland.

Corresponding author: Dr. Jacintha O’Sullivan, Trinity Translational Medicine Institute, Department of Surgery, St. James’s Hospital, Dublin 8, Ireland. Phone: +353-1-8962149, Email: osullij4@tcd.ie

**Supplementary Figure 1**

**Supplementary Figure 1: Quininib reduces the secretion of angiogenic growth factors and inflammatory cytokines in an explant model of colorectal cancer.**

Quininib reduced the secretion of ENA-78, GRO-α, TNF, IL-1β and MCP-1 in human colorectal tumor explants. Error bars are mean ± SEM. Statistical analysis was performed by ANOVA and Dunnett’s multiple comparison test.*: p<0.05, ***: p<0.001.

**Supplementary Figure 2**

**Supplementary Figure 2: Quininib does not reduce integrin fluorescence measured at day 31**

Fluorescence of α_v_β_3_ integrin-bound probe in animals treated with vehicle control, 5 mg/kg bevacizumab, 25 mg/kg quininib and 50 mg/kg quininib at day 31. There was no difference in fluorescence measurements between the treatment groups. Error bars are mean ± SEM.

**Supplementary Figure 3**

**Quininib**

**Bevacizumab**

PECAM1

ID3

ANGPT2

PROK2

ITGAV

STAB1

**THBS1**

TYMP

ITGB3

KDR

EGF

EFNA3

BAI1

SPHK1

**TGFBR1**

ID1

CCL11

FGF1

FLT1

CXCL10

IGF1

CDH5

NRP2

EREG

EFNA1

HIF1A

FGFR3

JAG1

TIMP3

HGF

IL8

IL6

PLG

FGF2

**VEGFC**

**PTGS1**

n=21 genes

n=10 genes

n=7 genes

CCL2

ANGPTL4

**Supplementary Figure 3 Quininib induces changes in gene expression in HT-29-luc2 cells**

Changes in gene expression in HT-29-luc2 cells when treated with quininib and bevacizumab. Gene names highlighted in bold indicated that these genes were up-regulated. Otherwise, all genes were down-regulated.
